# Starflo glaucoma implant: early experience in Hungary

**Published:** 2016

**Authors:** Cseke István, Vámosi Péter, Bausz Mária

**Affiliations:** * Elisabeth Academic Hospital, Sopron, Hungary; ** Péterfy Sándor Hospital and Traumatology Center, Budapest, Hungary; *** Department of Ophthalmology, Semmelweis University, Budapest, Hungary

**Keywords:** STARflo™, open angle glaucoma, suprachoroidal implant, glaucoma surgery

## Abstract

Aim: To present the early experience with the implantation technique, safety and efficiency of STARflo™ device for open angle glaucoma (OAG).

Methods: referring intra- and postoperative clinical experience with a series of seven cases in three glaucoma centers in Hungary.

Results: No intraoperative complications were observed. Postoperative inflammatory signs disappeared rapidly. The mean IOP reduction was from 27,6 ± 5,0 mmHg to 18,9±3,4 mmHg (32% reduction/ patient) at six months postoperatively.

Conclusion: STARflo™ implant was safe and (except for one case with neovascular glaucoma) effective in our cases. The learning curve for experienced anterior segment surgeons was short.

The prevalence of glaucoma together with the longer life expectancy is increasing. On the other hand, the interest for preserving the best possible quality of life is rising as well (for self- care, driving, and reducing the load of care of the society). These factors mean a demand for the development and research for the best- customized therapy of glaucoma by surgery as well.

The present study reports our experience with an implant routing the aqueous from the anterior chamber to the suprachoroidal space, hence enhancing the uveoscleral outflow.

This drainage mechanism was first explored with a surgical technique based on cyclodialysis introduced in 1905 by Leopold Heine. His original method, vehemently debated at first, has however been recognized all over the world later on, for treating open angle glaucoma and glaucoma in aphakic eyes. Various modifications of the primary surgical technique have been recommended, among them the combination of cyclodialysis with the other antiglaucomatous interventions as well as the implantation of tissue-components or foreign material into the cyclodialysis cleft. Apparently, a successful cyclodialysis resulted both in an increased aqueous outflow into the suprachoroidal space and in a reduced aqueous production. Nowadays, ab externo cyclodialysis has been abandoned due to the failure of the cleft to remain open and the increasing success of the other surgical techniques such as trabeculectomy [**1**]. However, cyclodialysis may still be helpful in the otherwise uncontrollable glaucomas and may regain a new importance with specific methods, as the one discussed in this article [**2**].

STARflo™ is a silicone implant for IOP reduction in open angle glaucoma (OAG). Its material is a flexible, tissue-friendly, micro- porous structure designed to reduce fibrotic response and maximize long-term performance. The intention of this study was to present the following: 1. Experiences of STARflo device implantation method. 2. Incidence of complications. 3. Reduction in IOP. 4. Reduction of glaucoma medication use.

## Method

STARflo™ device is made by iSTAR Medical in Belgium. Its medical grade silicone, controlled microporous geometric material (known as STAR® Biomaterial) is the result of ten years of research and development work undergone by the University of Washington and the Healionics Company. This material is designed to reduce fibrotic response and maximize long-term performance having tissue compatible properties to enhance bio integration. The STARflo V1 model (CE marked in 2012) is 11 × 6 mm with a head- neck-body design that helps preventing extrusion. In 2014, the Company issued an improved version of the device, STARflo v2. This latter version is slightly smaller with a more anatomical design of the head and a tapered body end to facilitate the introduction in the suprachoroidal space, while preserving the aqueous outflow capability. Principle of action: STARflo device enhances the aqueous flow through the natural uveoscleral path, without filtration bleb [**3**-**5**].

**Fig. 1:**
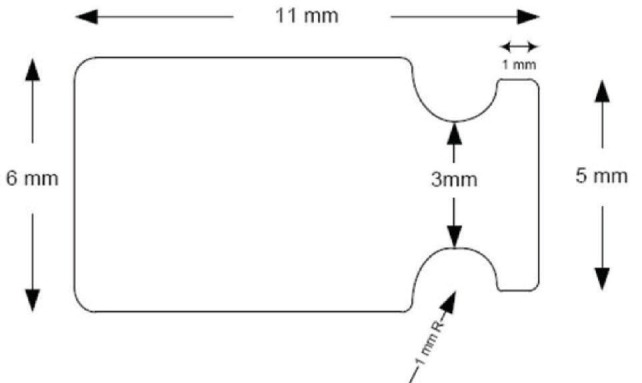
STARflo V1 model (source: iStar medical)

**Fig. 2:**
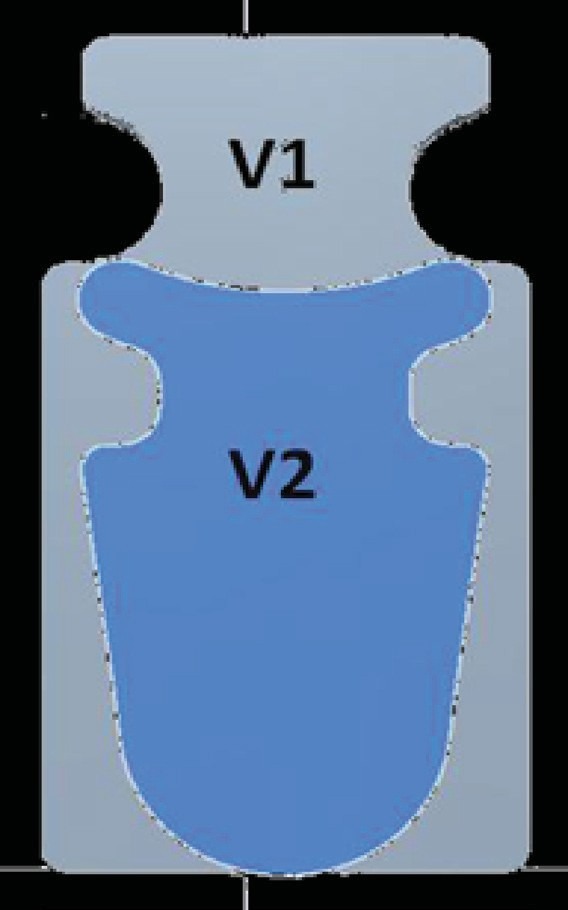
Comparison between the size of STARflo V1 and V2 model (source: iStar medical)

Surgical technique: The device is inserted through an ab externo approach into the suprachoroidal space via a scleral flap with its head positioned in the anterior chamber and the body of the device resting primarily between the sclera and choroid. The scleral flap is prepared parallel to the limbus (by the model V1: 3x8 mm). The sclera is penetrated completely on its base, and the suprachoroidal space is opened for the body of the implant. A 3.0 mm wide opening is created under the flap toward the anterior chamber and the head of the STARflo device is inserted through this sclerocorneal tunnel. The flap is then closed watertight over the implant and the conjunctiva is sutured as well.

**Fig. 3:**
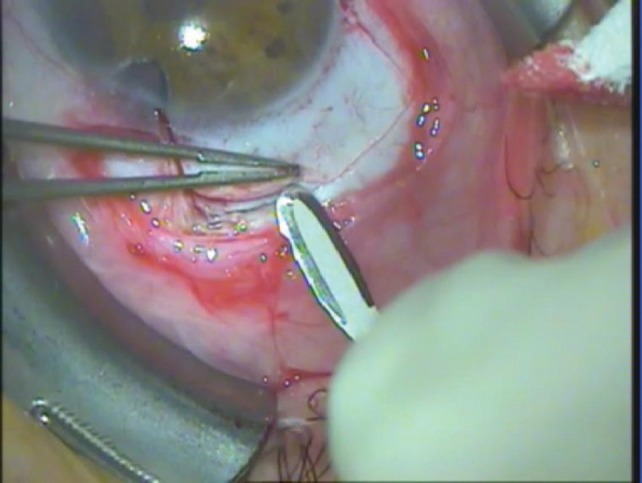
Preparing the half thickness scleral flap (photo by the author, Cs.I.)

**Fig. 4:**
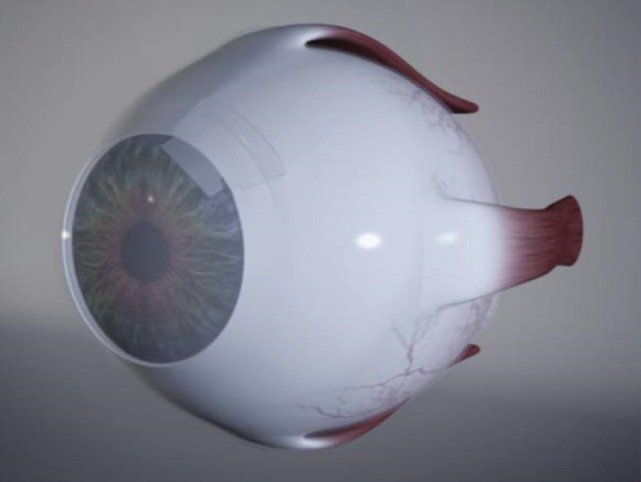
Position of the STARflo™ device after implantation (source: iStar Medical)

Patients: Since April 2013, the authors (from three centers in Hungary) implanted STARflo™ in seven patients (“V1” model in cases 1-3 and “V2” model in cases 4-7 (ages: 47 ± 8,5 years; four females, three males). Antifibrotic agents were not used.

Indication: All the seven patients had therapy resistant advanced glaucoma with former operations or trauma recorded in their medical history. There were four cases of a previous removal of a piece of the trabecular meshwork POAG, one case of traumatic glaucoma, and two cases of neovascular glaucoma.

## Results

In spite of the previous intraocular surgeries, intraoperative complications or severe inflammatory reactions were not observed. Moreover, postoperative inflammatory signs disappeared rapidly.

Later revisions were necessary in two cases: the repositioning of the head part had to be done by modifying the sclerocorneal tunnel wound, which appeared too long in these cases and caused endothelial touch and therefore circumscribed the corneal decompensation with epithelial instability (Case 1 - V1 and 4 - V2 model).

The mean preoperative IOP was 27,6 ± 5,0 mmHg and the mean preoperative glaucoma medication was 4,5 ± 1,7 intake/ day (6 cases). 1 month postoperatively, the mean IOP decreased to 23,4 ± 3,9 mmHg (15% average reduction/ patient) and the mean glaucoma medication decreased to 1.2 ± 1.7 intake/ day (6 cases). At 6 months, the mean IOP was 18,9 ± 3,4 mmHg (32% reduction/ patient) and the mean glaucoma medication was 2,3 ± 1,1 intake/ day (5 cases). No adverse events were reported during the surgery or immediately postoperatively (except for two transient hypotony, which proved to be harmless). No device-related serious adverse events were reported during the follow-up.

The early complications were the following: transient hypotony in 2 cases, transient choroidal detachment in 1 case.

**Fig. 5:**
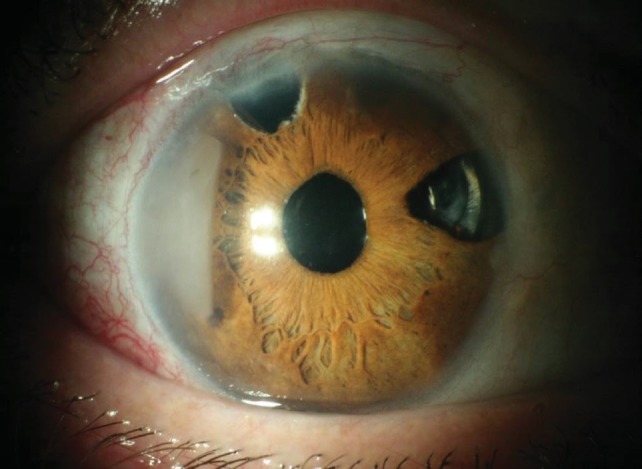
Case 1 (photo by the author, Cs.I.)

**Fig. 6:**
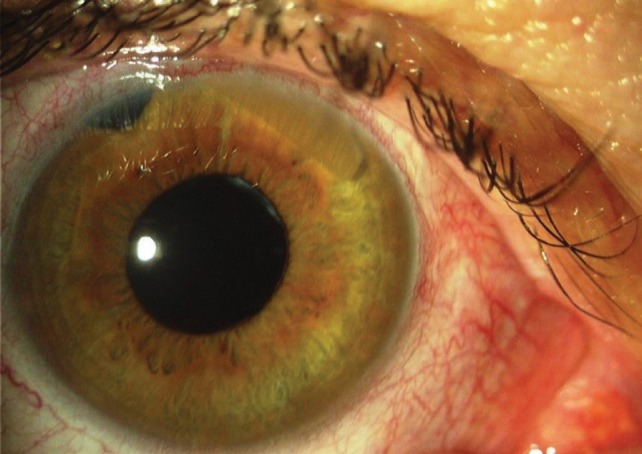
Case 2 (photo by the author, V.P

**Fig. 7:**
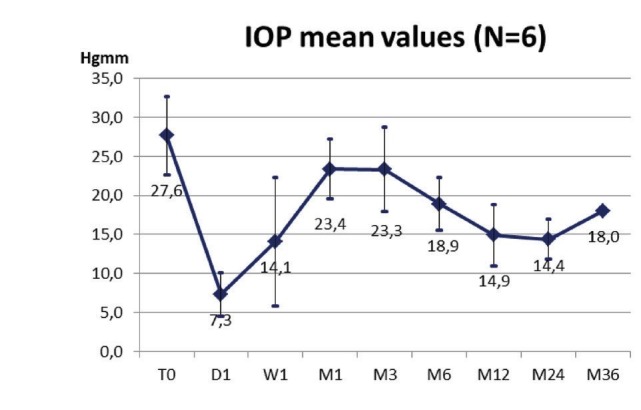
IOP data of the first experiences in the Hungarian centers (n=6)

## Discussion

Implants were used without complications in every operation. No removal was necessary. Our early postoperative complications were transient and mild, similar to those already reported in the previous publications (choroideal detachment, macular edema, mild hyphema, transient flat anterior chamber) [**3**,**4**]. Temporal choroidal detachment and two cases of transient hypotony were observed. The repositioning of the head part was performed in two cases (several months after surgery) because of the local discomfort due to the endothelial touch. A more careful wound construction is recommended to avoid this problem. The learning curve was relatively short, especially with the smaller and rounded V2 model. All the three surgeons had experience in cataract and glaucoma surgery, enhancing the scleral flap preparation and the implantation of the device. The surgical steps, which were thought to be most critical beforehand, were actually made without complications in every case: full thickness scleral penetration and preparation of the posterior suprachoroidal space. Theoretically potential difficulties such as choroidal bleeding or vitreoretinal complication were not observed. With its smaller size and rounded form, the new model (V2) will help performing this part of surgery even much easier. Based on these facts and on our experience, we believe that the learning curve is relatively short for surgeons having preliminary experiences in anterior segment surgery.

## Conclusion

Being aware of the previous successes of this implant in Europe, we selected rather difficult cases for our first patients. Our patients not only had OAG, but also multiple previous surgeries. The decompensated (proliferating) neovascular glaucoma case (case 4) did not prove to be a good candidate, the IOP remaining extremely high in this eye, already being blind in absolutum glaucoma state. On the other hand, by saving useful vision, our other neovascular glaucoma case (in regression phase, case 3), which was a last eye, can be regarded as a real success. No serious complications were observed in this complicated patient group. If we take out case 4 from our series, the reduction of the IOP and the number of the eye drop medication reduced significantly. Based on our experience, the implantation of STARflo™ device can be indicated in less advanced cases than in the cases of the patients we treated. In our opinion, the early results with STARflo™ device met expected safety and performance standards. The implant significantly reduces the IOP in refractory glaucoma and is safe. We observed eyes quickly regaining a peaceful state after the surgical intervention. Whether this method represents a real breakthrough in glaucoma surgery and could become a strong competitor for currently standard or innovative methods is to be supported by accumulated experience in several centers.

**Conflict of interest**

There is no financial interest. In five from the seven cases presented, the local distributor provided STARflo device for a free trial.

## References

[1] Samples Surgical Innovations in Glaucoma.

[2] Böke H (1990). History of cyclodialysis. In memory of Leopold Heine 1870-1940. Klin Monbl Augenheilkd.

[3] Pourjavan S (2013). STARflo glaucoma implant: 12 month clinical results. Acta Ophthalmol.

[4] Collignon N (2014). STARflo: A suprachoroidal drainage implant made from STAR biomaterial. Surgical Innova- tions in Glaucoma.

[5] Kammer JA, Mundy KM (2015). Suprachoroidal Devices in Glau- coma Surgery. Middle East Afr J Ophthalmol.

